# Gingival Verruciform Xanthoma: A Rare Case Report With Updated Insights Into Pathogenesis

**DOI:** 10.1155/crid/2174882

**Published:** 2026-07-14

**Authors:** J. Jeevarathan, S. Leena Sankari, Mohammed A. Assiri, S. Myvizhipriyadharshini, Siraj D. A. A. Khan, Khadijah Mohideen, Layla Hafed

**Affiliations:** ^1^ Department of Paediatric and Preventive Dentistry, Sree Balaji Dental College & Hospital, Bharath Institute of Higher Education and Research, Chennai, Tamil Nadu, India, bharathuniv.ac.in; ^2^ Department of Oral and Maxillofacial Pathology and Oral Microbiology, Sree Balaji Dental College & Hospital, Bharath Institute of Higher Education and Research, Chennai, Tamil Nadu, India, bharathuniv.ac.in; ^3^ Department of Oral and Maxillofacial Surgery and Diagnostic Sciences, Faculty of Dentistry, Najran University, Najran, Saudi Arabia, nu.edu.sa; ^4^ Jeeva′s Dental Care, Chennai, Tamil Nadu, India; ^5^ Department of Preventive Dental Sciences, Faculty of Dentistry, Najran University, Najran, Saudi Arabia, nu.edu.sa; ^6^ Deparment of Oral Medicine and Diagnostic Science, Faculty of Dentistry, Saba University, Sana′a, Yemen

**Keywords:** gingiva, oral mucosa, xanthoma

## Abstract

Verruciform xanthoma (VX) is a rare, reactive oral mucosal lesion that can mimic other papillary or verrucous hyperplastic conditions. The present report is of a gingival VX that clinically resembled other papillary lesions. A 14‐year‐old male reported a painless papillary lesion on the gingiva between the left mandibular central and lateral incisors, accompanied by erythematous anterior gingival margins. Based on clinical presentation, a provisional diagnosis of inflammatory gingival hyperplasia was considered. An excisional biopsy was performed, and histopathological examination revealed a hyperkeratotic, proliferative surface epithelium with elongated rete ridges and papillary projections filled with numerous foam cells, confirming the final diagnosis of VX. The patient has remained under follow‐up for 1 year with no evidence of recurrence. The present case highlights the importance of recognizing VX as a distinct entity in the diagnosis of oral papillary lesions. It discusses possible etiopathogenic factors associated with gingival involvement in adolescents.

## 1. Introduction

Oral verruciform xanthoma (OVX) is a rare, benign, hyperplastic papillary lesion primarily affecting the oral mucosa [1]. Other less frequently involved sites are the anogenital region and skin [2]. The pathogenesis remains unclear, but it is considered a benign reactive process, possibly triggered by local trauma, chronic irritation, or immune alterations [1]. OVX typically appears in individuals in their fifth or sixth decade [3]. Unlike cutaneous xanthomas associated with lipid disorders, OVX is not linked to systemic disease [4]. Its estimated prevalence ranges from 0.025% to 0.05% [5].

Clinically, OVX typically presents as a solitary, asymptomatic, slow‐growing lesion, often leading to confusion with papillary lesions. Differential diagnoses include viral warts, verruca vulgaris, verrucous hyperplasia, squamous papilloma, and early verrucous carcinoma [5]. OVX more commonly affects men, and the nodular papillary variant with a granular surface is more prevalent than the papular, plaque‐like, or ulcerative forms. [6] The most commonly affected sites are the gingiva, palate, buccal mucosa, tongue, vestibule, lip, and floor of the mouth [7, 8]. Preto et al. [9] stressed the need to monitor OVX in high‐risk areas like the tongue and floor of the mouth. This report presents a rare case of gingival OVX in an adolescent male and explores potential etiopathogenic factors related to gingival involvement.

## 2. Case Report

**Figure 1:**
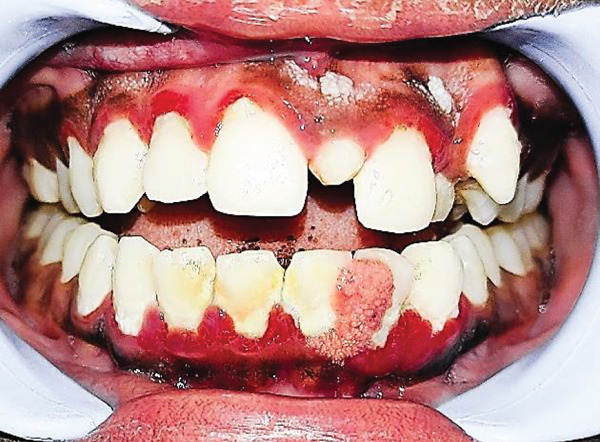
Intraoral picture shows exophytic growth between the lower left incisors.

A 14‐year‐old male presented with a 5‐month history of bleeding during brushing and a growth on the left lower anterior gingiva. He reported no pain, tobacco use, or any relevant medical or surgical history. Extraoral and systemic examinations were unremarkable. Intraorally, a pink, papillary exophytic lesion measuring approximately 1.2 × 0.7 cm was observed on the labial gingiva between the left lower incisors (Figure 1). The lesion was well‐demarcated, sessile, nontender, nonindurated, soft, asymptomatic, with a papillary surface and no associated cervical lymphadenopathy. The lesion′s history revealed that it began as a small papule and slowly enlarged to its current size, with no associated symptoms. Other clinical findings, such as generalized gingival inflammation, marginal erythema, mild bleeding, calculus deposits, mesiodens, and upper anterior crowding, were also noted. Overall periodontal health status was normal with no evidence of pockets. Heavy yellowish white supragingival calculus in the lower facial surface of anterior teeth and generalized hard subgingival calculus were seen. Based on the papillary nature and the related inflammation in the marginal gingiva, inflammatory gingival hyperplasia was considered the likely diagnosis. Other possible diagnoses included squamous papilloma, verruca vulgaris, condyloma acuminatum, and verrucous hyperplasia. Under 4% Articaine HCl with 1:100000 epinephrine as local anesthetic (Septanest, Septodont, Saint‐Maur‐des‐Fossés, France) in the lower anterior region, the lesion was excised using a diode laser (Ilase, Biolase Inc., Foothill Ranch, California, United States) with a preprogrammed setting for gingivectomy (1 W and pulse mode), and the specimen was submitted for histopathology. A complete oral prophylaxis was also performed over two visits.

**Figure 2:**
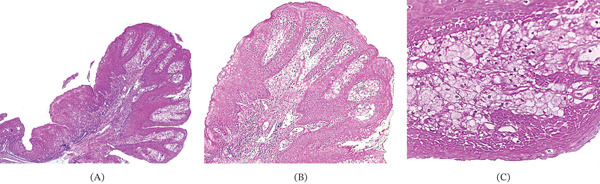
H&E picture shows (A) papillary epithelial hyperplasia in low power view (4x), (B) xanthomatous cells in the connective tissue papilla in low power view (10x), and (C) xanthomatous cells with the granular vacuolated cytoplasm in high power view (40x).

Histopathology revealed hyperparakeratinized stratified squamous epithelium with acanthosis, papillary projections, and parakeratin plugging (Figure 2A). Numerous xanthomatous cells with eccentrically placed flattened nuclei and granular cytoplasm were noted in the papillary connective tissue (Figure 2B,C), along with mild to moderate chronic inflammatory infiltrate and dilated capillaries in deeper connective tissue. Histopathological examination excluded other papillary lesions because viral cytopathic changes such as koilocytosis were absent, epithelial dysplasia was not identified, and the characteristic accumulation of numerous lipid‐laden foam cells within the connective tissue papillae beneath elongated rete ridges was present, confirming the diagnosis of verruciform xanthoma. The patient was monitored every 3 months after the surgery. Healing was successful, with no evidence of events, and the patient reported a reduction in inflammation. There were no postoperative complications, and at the end of the 1‐year follow‐up, the surgical site remained stable, with healthy gingival tissue and no signs of recurrence.

## 3. Ethical Approval and Informed Consent

Ethical approval was not required for this case report in accordance with the institution′s policies, as single‐case reports are generally exempt from institutional review board (IRB) review. Written informed consent for publication of the clinical details and any accompanying images was obtained from the patient′s parent. All identifying information has been anonymized to protect the patient′s privacy.

## 4. Discussion

VX predominantly occurs in the oral cavity, most often affecting the masticatory mucosa [1]. Less frequently, it involves other sites, such as the ventrolateral tongue and the floor of the mouth [5, 10]. In the present case, the lesion on the lower labial anterior gingiva aligns with Belknap et al.′s [1] findings of frequent gingival involvement. In our case, the lesion appeared pinkish‐white, whereas the literature describes a broader color range [7].

Clinically, OVX presents as a painless, sessile, or pedunculated exophytic papillary growth [3, 8, 11]. Most cases occur between the fifth and seventh decades; the present case is unusual due to its presentation in an adolescent patient [3, 7]. Pediatric and adolescent cases are extremely rare in the literature. A previously reported 14‐year‐old male patient had OVX involving the floor of the mouth [12]. In contrast, the present 14‐year‐old patient has a lesion on the lower anterior gingiva. Even though these cases are in different locations, they both showed no symptoms and had similar histopathological features, suggesting that the biological behavior of OVX is consistent across age groups. However, finding OVX in the gingiva of an adolescent is particularly unusual, as such lesions are more commonly seen in adults [1, 3]. Therefore, although the clinical and histopathological characteristics of the current case resemble those seen in adult OVX, its occurrence in an adolescent patient highlights how rare this lesion is in younger age groups.

The differential diagnosis for VX includes viral warts, squamous papilloma, verruca vulgaris, leukoplakia, verrucous hyperplasia, and early papillary squamous cell carcinoma [3, 8, 11]. The exophytic verrucous morphology of the lesion may be mistaken for a malignant process, emphasizing the need for careful histopathological evaluation.

Although the exact etiology and pathogenesis of OVX remain unclear, two main hypotheses have been proposed to explain lipid accumulation in lesional cells [4, 13]. The first hypothesis suggests that lipid accumulation in macrophages results from epithelial cell degeneration. As epithelial cells break down, the released lipids are engulfed by macrophages, which then form foamy cells. The common occurrence of OVX on masticatory mucosa supports this theory, linking chronic irritation, trauma, or inflammation to epithelial damage and lipid release [5]. However, this hypothesis is limited, as it does not explain OVX in trauma‐free areas, such as the soft palate or the floor of the mouth [7]. Additionally, histology often lacks evidence of epithelial degeneration, indicating an unclear pathogenesis.

An alternative theory proposes that foamy cells alter epithelial metabolism, leading to hyperkeratosis and the characteristic verrucous and papillary architecture. Some suggest the epithelial changes may be secondary to foamy macrophage accumulation or even “illusionary,” caused by upward pressure from these cells [5]. Both theories emphasize the importance of interactions among macrophages, keratinocytes, and chronic inflammation, as evidenced by the frequent presence of subepithelial inflammatory infiltrates in OVX cases.

The occurrence of OVX in both trauma‐prone and protected oral sites suggests that, beyond mechanical factors like trauma and irritation, chronic inflammation and localized immune responses also contribute to the development [3, 7]. Reported associations include food or metal allergies, tobacco and alcohol use, and certain medications [11]. It shows a possible link with chronic conditions such as gingivitis and periodontitis [3, 14]. In the present case, where mechanical irritation was unlikely, the lesion may be linked to an inflammatory condition like gingivitis. The present case suggests that local inflammatory factors may play an important role in its development, regardless of age. Additionally, OVX, unlike cutaneous xanthomas, is not associated with human papillomavirus infection [14].

Ide et al. [11] demonstrated that T cell‐mediated regulation via MCP‐1 and its receptor, CCR2, promotes macrophage recruitment to the subbasal papillae. Macrophages expressing MSR‐1 then engulf epithelial lipids within lysosomes. These studies reinforce the view of OVX as a slow‐growing, chronic reactive lesion.

VX has been reported in immunocompromised individuals and is associated with inflammatory conditions like lichen planus, discoid lupus erythematosus, graft‐versus‐host disease, and pemphigus vulgaris. OVX has also been linked to oral disorders such as amyloidosis, oral submucous fibrosis, leukoplakia, oral squamous cell carcinoma, and in patients undergoing chemotherapy or radiotherapy for lymphoma [3, 7, 9]. VX has been reported in patients with CHILD syndrome, involving a deficiency in an enzyme crucial for cholesterol synthesis [15]. Genetic studies have identified a missense mutation in Exon 6 of the NSDHL gene, which is essential for cholesterol biosynthesis, in 22% of cases of cutaneous xanthomas [14].

Histologically, verruciform xanthoma features a hyperkeratotic stratified squamous epithelium with verrucous or papillomatous growths and uniformly elongated rete pegs [7]. Parakeratin fills the clefts between epithelial projections [10]. A key characteristic is the presence of numerous foamy, lipid‐laden macrophages (xanthoma cells) within the connective tissue papillae between epithelial ridges. The connective tissue stroma usually shows mild to moderate chronic inflammatory infiltration and associated capillaries [1].

Nowparast et al. [13] classified VX into three histopathological types: Type A (warty/verrucous) with hyperparakeratosis, acanthosis, and elongated rete ridges; Type B (papillary or cauliflower) with finger‐like epithelial projections and connective tissue cores; and Type C (slightly raised or flat) showing mild acanthosis, variable rete ridge elongation, and thin parakeratosis. The present case was classified as papillary, exhibiting hyperplastic stratified squamous epithelium and connective tissue cores containing lipid‐laden macrophages. The most common differential diagnoses include squamous papilloma and verrucous carcinoma. Squamous papilloma demonstrates koilocytosis and lacks foamy macrophages, whereas verrucous carcinoma exhibits broad rete pegs composed of mature squamous epithelium with minimal atypia and also lacks foamy macrophages.

Immunohistochemical studies have confirmed that foam cells in OVX derive from the monocyte–macrophage lineage, showing strong cytoplasmic positivity for markers like CD68, cathepsin B, *α*1‐antitrypsin, and S‐100 [8, 14]. Shigeoka et al. (2020) further reported that CD168‐positive macrophages influence vascular endothelial growth factor expression [16].

Conservative surgical excision is the standard treatment for VX and yields an excellent prognosis [1, 3]. In this case, the lesion was removed using a diode laser, and no recurrence was reported after 1 year. Although rare, recurrences have been reported in sites like the lower lip, maxillary gingiva, and mandibular vestibule [1].

The present report is limited by its single‐case design, which precludes definitive conclusions regarding the etiopathogenesis and biological behavior of verruciform xanthoma in adolescents. Furthermore, the association between the accompanying gingival inflammation and lesion development remains speculative. Nevertheless, the rarity of gingival verruciform xanthoma in a 14‐year‐old patient and the absence of recurrence over 1 year of follow‐up provide valuable information in the limited literature on pediatric and adolescent cases.

## 5. Conclusion

OVX is a rare lesion with a distinctive histopathological pattern. Although its exact cause remains unclear, interactions between foamy histiocytes, epithelial cells, and chronic inflammation are key to its development. Accurate diagnosis relies on histopathology to differentiate it from similar verrucous lesions. This case report provides valuable clinical insights into VX pathogenesis.

## Author Contributions

J.J., L.S.S., and M.S.: concept and design and acquisition and interpretation of data. K.M. and L.H.: manuscript drafting and editing. M.A.A. and S.D.A.A.K.: critical review and final version.

## Funding

No funding was received for this manuscript.

## Disclosure

All authors have read and approved the final version of the manuscript. L.H. had full access to all data in this study and takes full responsibility for the integrity and accuracy of the data analysis.

## Consent

Patients provided written consent for the publication of their case details and accompanying images. A copy of the consent form is available upon request.

## Conflicts of Interest

The authors declare no conflicts of interest.

## Data Availability

The data that support the findings of this study are available from the corresponding author upon reasonable request.
